# Enhanced TDMA Based Anti-Collision Algorithm with a Dynamic Frame Size Adjustment Strategy for Mobile RFID Readers

**DOI:** 10.3390/s90200845

**Published:** 2009-02-06

**Authors:** Kwang Cheol Shin, Seung Bo Park, Geun Sik Jo

**Affiliations:** School of Computer and Information Engineering, Inha University, 253 Yonghyun-dong, Nam-gu, Incheon, 402-751, Korea; E-Mails: molaal@eslab.inha.ac.kr; gsjo@inha.ac.kr

**Keywords:** Mobile RFID reader, Anti-collision algorithm, Reader collision problem

## Abstract

In the fields of production, manufacturing and supply chain management, Radio Frequency Identification (RFID) is regarded as one of the most important technologies. Nowadays, Mobile RFID, which is often installed in carts or forklift trucks, is increasingly being applied to the search for and checkout of items in warehouses, supermarkets, libraries and other industrial fields. In using Mobile RFID, since the readers are continuously moving, they can interfere with each other when they attempt to read the tags. In this study, we suggest a Time Division Multiple Access (TDMA) based anti-collision algorithm for Mobile RFID readers. Our algorithm automatically adjusts the frame size of each reader without using manual parameters by adopting the dynamic frame size adjustment strategy when collisions occur at a reader. Through experiments on a simulated environment for Mobile RFID readers, we show that the proposed method improves the number of successful transmissions by about 228% on average, compared with Colorwave, a representative TDMA based anti-collision algorithm.

## 1. Introduction

Automatic identification (Auto-ID) refers to the methods of automatically identifying objects, collecting data about them, and entering that data directly into computer systems without human involvement. Auto-ID is an important technology in purchasing and distribution logistics, manufacturing companies and material flow systems [1]. Radio frequency identification (RFID) is a method of Auto-ID which involves remotely storing and/or retrieving data from small objects, so-called RFID tags. A tag, generally attached to an object, stores information about the object such as its serial number or cost [2-3].

Recently, Mobile RFID (MRFID) services have been introduced. MRFID can be defined as services that provide information on objects equipped with an RFID tag over a telecommunication network. The reader is installed in a mobile device such as a mobile phone, PDA, cart or forklift truck [4]. MRFID is different from the current implementations of ordinary RFID; here the readers are mobile and the tags are fixed, instead of the other way around. MRFID has some major and obvious advantages over RFID; wires to the fixed readers are no longer needed and several mobile readers are often sufficient to cover a whole area, instead of requiring dozens of fixed readers [5].

MRFID can be applied in various areas. In a warehouse, an RFID reader mounted on a forklift truck reads the tags of the loads on the pallet and automatically identifies the information on each load such as ‘what it is’ and ‘where it is going/coming from’ without having to take the loads off the pallet. Another interesting application in a supermarket would be where a reader attached to a shopping cart displays a list of items within its range, so that the customer need not look through the shelves in search of the desired items. MRFID can also be applied in libraries. Handheld readers can have customized client side applications such as searching for a particular book, or counting the number of items on the shelf [4].

However, to use MRFID in real situations, we should consider the potential collision problems between the readers as they attempt to read the tags, since the readers are continuously moving. There are two situations where reader collisions can occur; reader to reader interference and multiple reader to tag interference [6]. Reader to reader interference arises when the stronger signals from a reader interfere with the weak reflected signal from a tag, while multiple reader to tag interference arises when more than one reader tries to read the same tag simultaneously [7].

In this study, we propose an enhanced Time Division Multiple Access (TDMA) based anti-collision algorithm to solve these reader collision problems. In our algorithm, when a reader collision occurs at a reader, the reader changes its number of timeslots of a frame according to the number of readers with which it can interfere. As an experiment, we build a simulated MRFID environment in which we consider the mobility of the readers. Through experiments, we show that the proposed method can improve the number of successful transmissions corresponding to the reading of tags by 228%, compared with Colorwave [8-9], a representative TDMA based anti-collision algorithm.

This study is organized as follows: we review existing anti-collision solutions in Section 2 and explain the proposed algorithm in Section 3. In Section 4, we explain the simulated test environment for the Mobile RFID and the experimental results are given in Section 5. Concluding remarks and further research directions are explained in Section 6.

## 2. Literature Review on Anti-collision Algorithms

Anti-collision algorithms can be categorized as either Frequency Division Duplexing (FDD), Carrier Sense Multiple Access (CSMA), or Time Division Multiple Access (TDMA) [7] and we will explain each of these categories briefly in this section.

### 2.1. FDD based anti-collision algorithms

To solve the reader collision problem, RFID standards such as ISO/IEC 18000-6 [10] and EPC Class 1 Generation 2 [11] basically use spectral planning (FDD). This method separates the reader transmissions and the tag transmissions spectrally, such that tags collide with tags but not with readers and readers collide with readers but not with tags. Such a separation solves the reader to reader interference problem, since the reader transmissions and tag transmissions are on separate frequency channels. However, this approach does not solve the reader to tag collision problem when more than two readers simultaneously try to read the same tag, because passive tags of UHF bandwidth can only reflect the signals from the readers to transmit their information. Thus, multiple reader to tag interference still exists in these standards.

### 2.2. CSMA based anti-collision algorithms

ETSI EN 302 208 [12] has a CSMA based protocol called “Listen Before Talk” to prevent reader collisions. The reader first listens on the data channel for any on-going communication for a specific minimum time. If the channel is idle for that time, it starts reading the tags. If the channel is not idle, it chooses a random backoff. However, ETSI EN 302 208 still leaves the door open to reader collisions when the readers are not within each other's sensing range (hidden terminal); readers that are not within each other's sensing range may interfere with each other when they try to read the tags.

Birari and Lyer [13] suggested a simple Pulse protocol which is based on periodic beaconing on a separate control channel by a reader, while the reader is reading the tags. They showed that their algorithm reduces the reader collision rate to 1-2% and also increases the read rate to as high a value as 98% by comparing it with ETSI EN 302 208. Their algorithm works on the assumption that a reader is able to simultaneously receive on both the control and the data channel.

### 2.3. TDMA based anti-collision algorithms

In TDMA based anti-collision algorithms, each reader divides a frame into several timeslots and reads the tags using one of these timeslots in a frame, so that reader to reader interference and multiple reader to tag interference can be prevented.

#### 2.3.1. DCS and Colorwave

As a TDMA based anti-collision algorithm, Distributed Color Selection (DCS) [8-9] allows the easy reservation of a timeslot; a color is used to represent a periodic reservation for the collision-free transmission of data. A reader with a queued request for transmission transmits only in its color (timeslot). If the transmission collides with that of another reader, the transmission request is cancelled and the reader randomly chooses a new color and reserves this color. ‘Reserve’ means that if a neighboring reader has the same color, it has to choose another color, thereby clearing the timeslot for the reader the next time around. This switch and reservation action is referred to as a ‘kick’. The maximum number of colors is input to the algorithm, and does not change throughout its execution. Each reader keeps track of what color it believes the current timeslot to be. DCS is a “greedy” algorithm - a node's chances of colliding immediately after experiencing a collision are minimized at the expense of its neighbors.

Since in DCS the maximum number of colors is fixed, a mechanism for dynamically changing the maximum number of colors available at a reader is needed. Thus, Colorwave [8-9] was suggested as an advanced version of DCS. In Colorwave, each reader chooses a random color from 0 to max_colors to transmit. If it collides, it selects a new timeslot and sends a kick (small control packet) to all of its neighbors to indicate the selection of a new timeslot. If any neighbor has the same color, it chooses a new color and sends a kick and so on. Each reader keeps track of what color it believes the current timeslot to be, as in the case of DCS. However, in Colorwave, each reader monitors the percentage of successful transmissions. Five manual parameters determine when a reader changes its local value of max_colors:

- UpSafe: The safe percentage at which to increase max_colors.
- UpTrig: The trigger percentage at which to increase max colors, if a neighboring reader is also switching to a max_colors, if a neighboring reader is also switching to a max_colors higher than that of this reader.
- DnSafe, DnTrig: analogues of UpSafe, UpTrig, except decreasing max_colors.
- MinTimeInColor: The minimum number of timeslots before the Colorwave algorithm will change max_colors again after initialization or changing max_colors.

When a reader executing Colorwave reaches a Safe percentage to change its own value for max_colors, it will send out a kick to all of its neighboring readers. If the phenomenon that is causing it to exceed a Safe percentage is local to that reader, the other readers will not have passed their own Trig percentages and will not respond. However, if the phenomenon causing the collision value to exceed a Safe threshold is widespread, neighboring readers will most likely have exceeded their own Trig thresholds, and a kick wave will ensure. As kicks spread from the initiating reader throughout the entire system, a large portion or all of the readers in the system may change their value of max_colors. Colorwave works well in the situation where the RFID readers are fixed or barely move.

#### 2.3.2. Timeslot structure of TDMA based anti-collision algorithms

**Figure 1.**
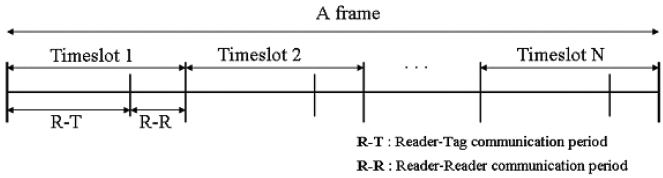
Frame structure of TDMA based anti-collision algorithm.

In TDMA based anti-collision algorithms, a reader to reader communication period should exist, because each reader decides its timeslot for transmission through communications between the readers. Figure 1 shows a frame which consists of timeslots and each timeslot is divided into Reader to Tag (R-T) and Reader to Reader (R-R) communication periods. The R-T period is used for reading the tags of a reader and the R-R period is used for the communications between readers. Our algorithm also follows this frame structure.

## 3. Proposed Anti-collision Algorithm for Mobile RFID Readers

### 3.1. Description of the algorithm

In this section, we describe our algorithm for Mobile RFID, where ‘Mobile’ means that each RFID reader can move anywhere. Colorwave is appropriate in the situation where the RFID readers are fixed or barely move. However, in the case of Mobile RFID readers, since the readers keep moving, reader collisions can occur frequently and so the value of max_colors becomes unnecessarily high, especially in the case where the readers gather together densely in a small area. Our algorithm automatically controls the number of timeslots allocated to a reader in a frame by setting its value according to the number of readers, which are located in the interfere range of the reader.

**Figure 2.**
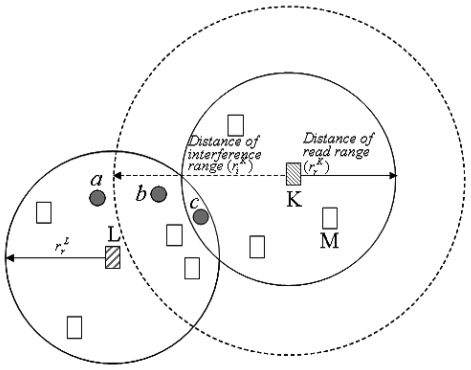
Example of reader collision.

Figure 2 shows the three possible locations of tags, which are represented by gray circles, to be read by reader *L* (Readers are represented by rectangles). There is no problem in reading the tag *a*. However, in case of the tag *b*, the signal returned from tag *b* is interfered by the signal of reader *K*, if reader *K* sends signal to read tags at that time. The tag *c*, which is located in the read ranges of reader *K* and *L*, brings interference problem to both reader *K* and *L* if two readers attempt to read the tag at the same time. Besides of reader to tag interference problem, reader to reader interference can occur if reader *K* and *M*, which are located in the read range of each other, try to read at the same time. To deal with these interference problems, we design an algorithm as shown in Table 1.

Our algorithm consists of three parts and works in a wholly distributed manner for each reader. In the first part, each reader waits until its timeslot for transmission occurs. In the second part, a reader which has suffered a collision changes the number of timeslots in a frame (max_timeslots) to

$$\alpha +\left\lfloor \alpha \times \frac{\left(r_{i}^{2}-r_{r}^{2}\right)}{r_{r}^{2}}\right\rfloor +1$$

where *α* is the number of readers, which are located in its read range, *r_i_* and *r_r_* mean the distance of interference range and read range of the reader respectively.

**Figure 3.**
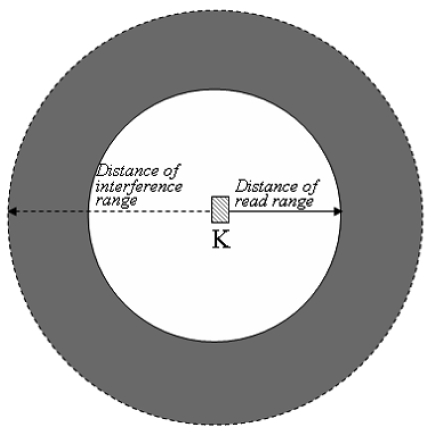
Interference and read range of a reader.

We form the formula for max_timeslots of the reader by the reason that a reader can count the number of readers which are located in its read range but has to estimate the number of readers which are located in the area of interference range outside of the read range (Gray area of figure 3). And ‘+1’ for counting the reader itself.

To count the number of readers in the read range of reader *K*, reader *K* broadcasts a message with its id in R-R period of timeslot as shown in Figure 1, and the readers which receive the message return it to the sender and consequently reader *K* perceives the number of readers in its read range by counting the number of return messages. We implemented the tree algorithm [14], which is used in a reader to read multiple tags without collision, to receive the return messages from multiple readers without collision.

The reader randomly selects a timeslot for transmission (current_timeslot) within its max_timeslots range and broadcasts the current_timeslot with clock synchronization signal to the neighboring readers which are located in read range.

In the third part, if a reader receives a broadcast message and did not send any broadcasting messages so far in current frame, then it set the clock to be synchronized with the broadcaster to start the next timeslot in the same time with sender and it randomly changes its current_timeslot to avoid collisions with the broadcasting reader in the next frame. Communications with neighboring readers also occur in the Reader to Reader communication period in a frame, as shown in Figure 1.

To facilitate the understanding of our algorithm, we explain the algorithm by using an example in Figure 2 with assuming that the distance of each reader's read range is 2 meter and the distance of interference range is 3 meter. When reader *K* tries to read the tag *c*, but detects a collision with one of the readers in its interference range (in this case, we assume that reader *L* interferes with reader *K*), reader *K* changes its number of timeslots in a frame to 8 by calculating 3+⌊3×(3^2^−2^2^)/2^2^⌋+1. At the same time, because reader *L* also experiences a collision, it also changes its number of timeslots in a frame to 10 by calculating the formula. After changing their numbers of timeslots, readers *K* and *L* randomly select their timeslot (current_timeslot) for reading tags in the next frame and let the other readers, which are located in their interference ranges, know their selection of current_timeslot by broadcasting the information. If a reader, except for the readers that are broadcasting (in this case, readers *K* and *L*), receive information including a new current_timeslot, it randomly selects its new current_timeslot within the limit of its max_timeslot. Table 1 shows the whole proposed algorithm. Our algorithm supports the full automatic self adjustment of the frame size of each reader separately according to its number of neighbors.

### 3.2. Analysis of the algorithm

If number of *n* readers, whose interference distance is *r*, is activated in the *M* by *N* rectangular shape of target area during *δ* time period, the properties of a reader *K*, which adopts our algorithm, can be modeled as follows:

- The estimated number of readers, which located in the range of interference with a reader *K*, is:
  $$n\times \frac{\pi r_{k}^{2}}{MN},\;\text{where}\;\pi \;\text{is the circular constant}$$
- A frame size of a reader *K* according to our algorithm is:
  $$\frac{n\pi r_{k}^{2}}{MN}+1$$
- The probability of reader collision in a reader *K* is:
  $$\frac{n\pi r_{k}^{2}/MN}{(n\pi r_{k}^{2}/MN)+1}$$
- The probability of continuous reader collision during *δ* time period in a reader *K* is:
  $$\frac{n\pi r_{k}^{2}/MN}{(n\pi r_{k}^{2}/MN)+1}\times \frac{\delta }{(n\pi r_{k}^{2}/MN)+1}=\frac{\delta n\pi r_{k}^{2}MN}{{\left(n\pi r_{k}^{2}+MN\right)}^{2}}$$
- The maximum number of successful transmissions of a reader *K* during *δ* time period can be:
  $$\frac{\delta }{(n\pi r_{k}^{2}/MN)+1}=\frac{\delta MN}{n\pi r_{k}^{2}+MN}$$
- The minimum number of successful transmissions of a reader *K* during *δ* time period can be:
  $$\frac{\delta MN}{n\pi r_{k}^{2}+MN}-\frac{\delta n\pi r_{k}^{2}MN}{{\left(n\pi r_{k}^{2}+MN\right)}^{2}}=\frac{\delta MN}{n\pi r_{k}^{2}+MN}\times (1-\frac{n\pi r_{k}^{2}}{n\pi r_{k}^{2}+MN})$$

According to above formula, our algorithm guarantees the minimum number of successful transmissions of a reader. (The results of the number of successful transmissions in figure 9 of the section 5 match with the above formulas)

## 4. Environment for Experiments

**Figure 4.**
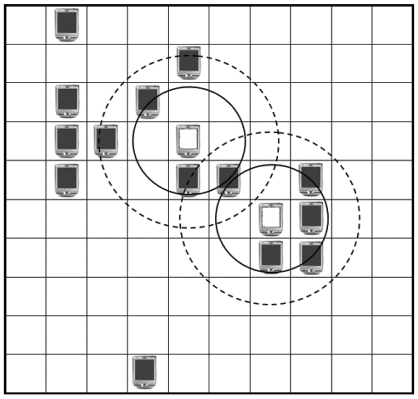
Interference and read range of a reader.

For the experiments on the anti-collision algorithm for Mobile RFID, a simulated 10 by 10 virtual matrix where each cell size is 4 meters by 4 meters was implemented and, in this matrix, the readers had mobility to move anywhere. The simulator was built on the assumption that each RFID reader uses ultra high frequency (UHF) 868-960 MHz whose distance of read range is 3 to 7 meters, according to the signal strength with zero packet loss. We set the distances of read and interference range of each reader as 6 and 8.5 meters respectively such that it could communicate with its eight neighboring readers and interferes with 17 readers in maximum, as shown in Figure 4.

**Figure 5.**
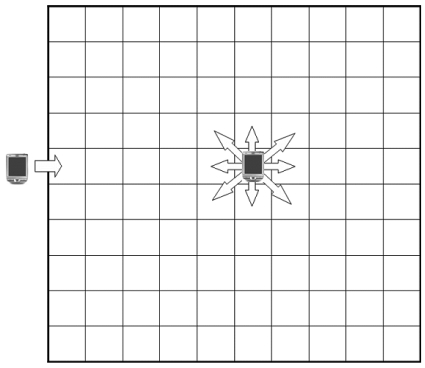
A reader's entering the matrix and its moving direction.

Our scenario for the experimental environments is as follows: at first, there is no reader present in the matrix and, at timeslot_ID 1 (the timeslot_ID represents the elapsed time and increases from 1 to 100,000), a reader enters into one of the boundary cells of the matrix randomly, as shown in Figure 5. As the timeslot_ID increases one by one, readers enter into the matrix one by one until the desired maximum number of readers is attained. Once a reader enters into the matrix, it can move to one of its 8 neighboring which is not occupied by any other reader. We set the parameters so that one third of the readers, which are randomly selected, move randomly to an empty neighboring cell every 10 timeslot_IDs.

## 5. Experimental Results

For the experiments, we implemented the simulation environment and anti-collision algorithm using Java on the Windows® XP platform of the Intel^®^ Pentium^®^ Dual CPU 3.40 GHz with 896 MB RAM. To test our algorithm in various environments, we adjusted the number of readers which are inserted into the matrix to 10 for sparse, 20 for medium, and 30 for a dense distribution of the readers. We compared our algorithm with Colorwave because the latter is the representative TDMA based anti-collision algorithm, as mentioned in Section 2. We adopt four factors to compare the performances of Colorwave and our algorithm, AC_MRFID, viz; Frame Size, Frame Utilization, Broadcasting Messages, and Successful Transmissions, as defined in Table 2. We set the manual parameters of Colorwave to 90 for UpTrig, 93 for UpSafe, 99 for DnTrig, 98 for DnSafe, and 100 for MinTimeInColor, as defined in the following references [8-9]. Each experiment was repeated 10 times and the average values of each comparative factor were obtained every 100 timeslot_IDs. We set the initial frame size of each reader to one in both Colorwave and our algorithm.

**Figure 6.**
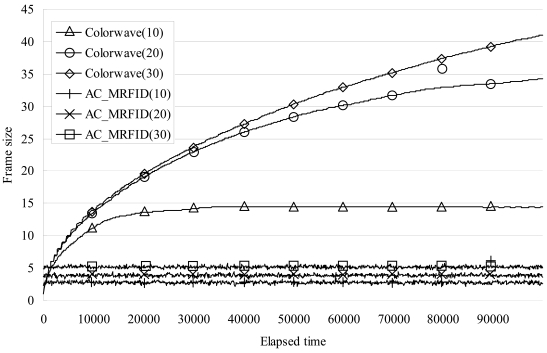
Comparison of results of average frame size of readers.

Figure 6 shows the average frame size of the readers per 100 elapsed timeslot_IDs (The marks on the graph are at every 10,000 elapsed timeslot_IDs for the sake of clarity).

As the figure shows, when the test was conducted with 10 readers, the average frame size of Colorwave approached 14.5, while that of our algorithm remained stable at 2.5. With 20 readers, the frame size of Colorwave increased to 34, while that of our algorithm was stable at 4. With 30 readers, the frame size of Colorwave went up to 41, while that of our algorithm remained at 5.2. This results show that the manual parameters of Colorwave needs to be adjusted carefully according to the density of the readers, while our algorithm automatically adjust the frame size without the need for manual parameters.

**Figure 7.**
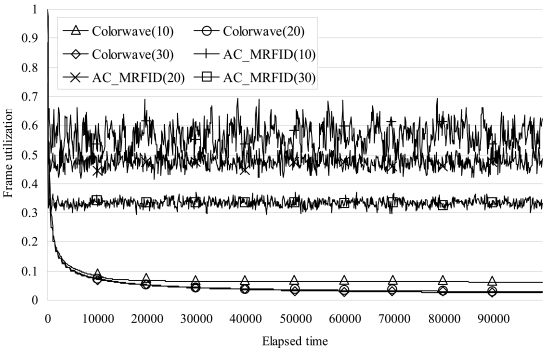
Comparison of results of frame utilization.

Figure 7 shows the frame utilizations in each of the three cases for Colorwave and AC_MRFID. The frame utilization means the ratio of the number of slots used for transmission in a frame. If it approaches 1, it means that all of the timeslots of a frame were used for transmissions without any wasted timeslots. As the figure shows, when the test was conducted with 10 readers, the frame utilization value in the case of our algorithm varied from 0.88 to 0.42, while that of Colorwave gradually decreased to 0.06. In the case of 20 readers, our algorithm varied from 0.74 to 0.41, while that for Colorwave approached 0.029. With 30 readers, our algorithm varied from 0.69 to 0.29, while that for Colorwave approached 0.024.

**Figure 8.**
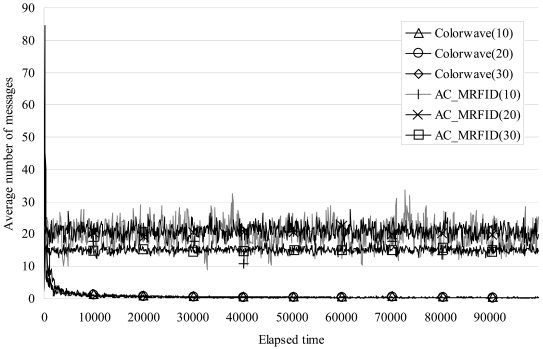
Comparison of results of average number of messages.

Figure 8 shows the results of average number of broadcasting messages of a reader for reader to reader communication per 100 elapsed timeslot_IDs. As the figure shows, when the test was conducted with 10 and 20 readers, a reader, which adopted AC_RFID algorithm, continuously sent broadcasting messages about 20 times on average per 100 elapsed timeslot_IDs, and with 30 readers, about 15 times on average, while a reader, which adopted Colorwave algorithm, sent broadcasting messages over 80 times in the first 100 timeslot but the number drastically decreased to 0.5 times as times went on in all 3 cases. This results show that AC_RFID algorithm make a reader enable to keep communicate each other continuously to decide its number of timeslots in a frame appropriately. In the sense of energy consumption, though AC_RFID consumed more energy than Colorwave to communicate each other, it would not be a big problem not as like scattered sensor nodes because generally RFID reader devices have ample capacity of batteries which can be recharged as like cell phone or PDA device.

**Figure 9.**
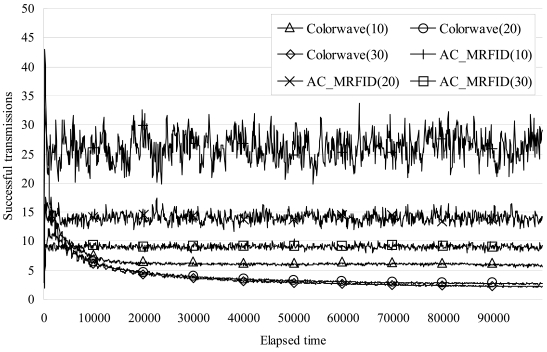
Comparison of results of successful transmissions.

Finally, Figure 9 shows the average number of successful transmissions of each reader without collision per 100 elapsed timeslot_IDs. In the case of 10 readers, our algorithm, AC_MRFID, successfully transmitted between 19 and 33 times. On the other hand, in the first 500 timeslots Colorwave transmitted with a higher frequency than AC_MRFID, but, as time went by, the value decreased to 5.5 times. In the case of 20 readers, our algorithm successfully transmitted between 5 and 22 times, while Colorwave showed better results in the first 1000 timeslots, but as time went by, its value decreased to 2.6. In the case of 30 readers, our algorithm successfully transmitted between 8 and 10 times, while Colorwave showed better results in the first 4400 timeslots but, as time went by, its value decreased to 2.2. The overall average numbers of successful transmissions in both cases are listed in table 3. As Table 3 shows, our anti-collision algorithm for Mobile RFID improves the number of successful transmissions by 228.4% on average compared with Colorwave.

## 6. Conclusions

Mobile RFID is an emerging technology which can be used in supermarkets, warehouses or libraries for the purpose of searching for items or automatically checking them in or out. In this study, we proposed a TDMA based anti-collision algorithm for RFID readers. We verify that our algorithm produces a stable frame size for each reader and provides a better number of successful transmissions for each reader, regardless of the density of the Mobile RFID reader distribution even without using manual parameters. Based on the experimental results, we expect that our anti-collision algorithm can be applied in various industrial fields and we plan to implement it in real Mobile RFID readers.

## Figures and Tables

**Table 1.** Pseudo code of proposed algorithm.

**Transmission::**
if (timeslot_ID% max_timeslots)==current_timeslot
then transmission
else idle until (timeslot_ID%max_timeslots)==current_timeslot
**Collision::**
let *α* as the number of readers which are in read range
let *r_i_* and *r_r_* as the distances of interference and read range respectively
if attempted transmission but experienced collision
$\text{then max}\_\text{timeslots}=\alpha +\left\lfloor \alpha \times \frac{\left(r_{i}^{2}-r_{r}^{2}\right)}{r_{r}^{2}}\right\rfloor +1$
current_timeslot=random(max_timeslots)
broadcast the information stating current_timeslot with synchronization signal
**Collision Resolution::**
if the information received stating current_timeslot if I didn't broadcast in this frame
then set clock to be synchronized with broadcaster's
current_timeslot=random(max_timeslots)

**Table 2.** The comparative factors.

**Comparative Factors**	**Definitions**
Frame Size	Average frame size of readers per unit time
Frame Utilization	Average ratio of used slots in a frame of readers per unit time (used slots in a frame/the frame size)
Broadcasting Messages	Average number of broadcasting messages of a reader for reader to reader communication per unit time
Successful Transmissions	Average number of transmissions without collision of readers per unit time

**Table 3.** Overall average numbers of successful transmissions.

**Number of Readers**	**AC_MRFID**	**Colorwave**	**Improvements** **((AC_MRFID-Colorwave)/Colorwave)**
10	26.14	6.69	290.6%
20	14.01	4.05	245.7%
30	9.03	3.63	148.9%
**Average**	**16.40**	**4.79**	**228.4%**
